# Ecological, Evolutionary and Social Constraints on Reproductive Effort: Are Hoary Marmots Really Biennial Breeders?

**DOI:** 10.1371/journal.pone.0119081

**Published:** 2015-03-13

**Authors:** Vijay P. Patil, Timothy J. Karels, David S. Hik

**Affiliations:** 1 Department of Biology and Wildlife, University of Alaska Fairbanks, Fairbanks, Alaska, 99775, United States of America; 2 Department of Biological Sciences, University of Alberta, Edmonton, AB, T6G 2E9, Canada; University of Manitoba, CANADA

## Abstract

Biennial breeding is a rare life-history trait observed in animal species living in harsh, unproductive environments. This reproductive pattern is thought to occur in 10 of 14 species in the genus *Marmota*, making marmots useful model organisms for studying its ecological and evolutionary implications. Biennial breeding in marmots has been described as an obligate pattern which evolved as a mechanism to mitigate the energetic costs of reproduction (Evolved Constraint hypothesis). However, recent anecdotal evidence suggests that it is a facultative pattern controlled by annual variation in climate and food availability (Environmental Constraint hypothesis). Finally, in social animals like marmots, biennial breeding could result from reproductive competition between females within social groups (Social Constraint hypothesis). We evaluated these three hypotheses using mark-recapture data from an 8-year study of hoary marmot (*Marmota caligata*) population dynamics in the Yukon. Annual variation in breeding probability was modeled using multi-state mark-recapture models, while other reproductive life-history traits were modeled with generalized linear mixed models. Hoary marmots were neither obligate nor facultative biennial breeders, and breeding probability was insensitive to evolved, environmental, or social factors. However, newly mature females were significantly less likely to breed than older individuals. Annual breeding did not result in increased mortality. Female survival and, to a lesser extent, average fecundity were correlated with winter climate, as indexed by the Pacific Decadal Oscillation. Hoary marmots are less conservative breeders than previously believed, and the evidence for biennial breeding throughout *Marmota*, and in other arctic/alpine/antarctic animals, should be re-examined. Prediction of future population dynamics requires an accurate understanding of life history strategies, and of how life history traits allow animals to cope with changes in weather and other demographic influences.

## Introduction

Animals living in harsh, unpredictable environments may require unusual life history traits to maximize their fitness. Biennial breeding, or reproductive skipping, is one such trait that is found in only a few groups of long-lived birds and mammals in polar and alpine habitats [1–5]. Biennial breeding is generally explained as a way of maximizing lifetime reproductive output when frequent reproduction is energetically costly and dangerous [2,5–7]. However, harsh environments can also be highly stochastic [8,9]. In such situations, a conservative life history involving biennial breeding may reduce individual fitness, because animals that skip one reproductive opportunity may not live long enough to reproduce in the future [10].

The best documentation for biennial breeding patterns comes from long-lived seabird species with small clutch sizes [1,2,5]. For example, biennial breeding is commonly reported in albatross species, many of which can live for 40 years or more and lay only one egg at a time [5]. Reproductive skipping in albatrosses may be beneficial for these species because their life histories prioritize parental investment in a very limited number of young, and because their long lifespans allow them ample breeding opportunities even if they do not attempt to breed every year [5]. However, it remains unclear why biennial breeding persists in species with short lifespans and variable fecundity.

Among mammals, biennial breeding (a consistent pattern of biennial reproduction with few or no examples of annual reproduction by individuals) is reportedly common in the genus *Marmota*. Of the fourteen extant marmot species, ten appear to skip one or more years when breeding [4]. As with biennial-breeding birds, most marmot species live in arctic or alpine environments with harsh but variable weather [4]. Marmots are also relatively long lived and philopatric, making them a convenient choice for long-term population-level studies of breeding. Three alternative but not mutually exclusive hypotheses could explain biennial breeding in marmots: the Evolved Constraint hypothesis, Ecological Constraint hypothesis, and the Social Constraint hypothesis.

The Evolved Constraint hypothesis states that biennial breeding in marmots is a canalized evolved pattern dictated by the physiological costs of reproduction in a harsh environment (e.g. energy and fat consumption) [11]. Evolved constraints could have arisen if a single growing season is inadequate for females to recover the energy and fat expended during reproduction, limiting the ability of annual breeders to survive hibernation and ultimately reducing their fitness relative to biennial breeders. This hypothesis leads to the prediction that annual breeding should be extremely rare or absent [12]. If marmots are subject to evolved constraints on reproduction, breeders are also likely to have reduced survival compared to nonbreeders during the next winter, although an association between survival and breeding history is not sufficient proof of evolved constraints in itself. Breeding female hoary marmots (*Marmota caligata*) and Olympic marmots (*Marmota olympus*) spend more time foraging, stay active later in the fall, and weigh less at immergence than nonbreeders, which demonstrates that there is a physiological (energetic) cost of breeding for marmots [13,14]. However, it is not clear whether breeding and survival are negatively associated in either species.

Both Olympic and hoary marmots are considered biennial breeders, but annual breeding has been anecdotally observed in both species [15,16]. This leads to the Ecological Constraint hypothesis, which is that biennial breeding may occur when the animal does not have enough energetic capital or cannot acquire sufficient energetic income to support more frequent reproduction [17]. For capital breeders, this hypothesis leads to the prediction that marmots that bred in the previous year should have a reduced probability of breeding in the current year due to the physiological costs of reproduction [13]. Ecological constraints due to a capital breeding strategy would also be consistent with a prediction of reduced survival probability for breeding females compared to nonbreeders. If hoary marmots are income breeders, the Ecological Constraint hypothesis predicts that the probability of breeding should be a function of environmental conditions that dictate food availability, such as the timing of spring snowmelt [17]. These predictions are not mutually exclusive, depending on where hoary marmots fall along the Capital-Income breeding continuum.

Because marmots are social animals, biennial breeding may also be caused by competition between females (Social Constraint hypothesis). Dominant females suppress reproduction by subordinates in both alpine marmot (*Marmota marmota*) and yellow-bellied marmot (*Marmota flaviventris*) social groups [18,19]. In alpine marmots, this behavior leads to higher survival among the dominant female’s offspring, which benefit from the presence of non-breeding subordinate adults during hibernation [20]. Most biennial breeding marmot species are thought to practice reproductive suppression, but suppression has been difficult to document in wild populations [6,15]. The Social Constraint hypothesis leads to the prediction that the average probability of breeding should decrease as the number of mature females per group increases [21]. Reproductive suppression may also result in decreased breeding probability for younger females, especially in larger groups [18].

We studied the breeding and survival probabilities of hoary marmots in the Yukon, Canada using multi-state mark-recapture models [22,23]. Hoary marmots occur in high-latitude and alpine habitat with harsh, unpredictable weather, and their social groups exhibit highly variable social structure, which makes them a uniquely appropriate model for testing the Evolved Constraint, Ecological Constraint, and Social Constraint hypotheses [16,24]. Our objectives were to determine the probability of annual and biennial breeding, and to evaluate the relative likelihood of our three hypotheses. We also examined sources of variation in fecundity to determine whether the processes that governed breeding probability also influenced other reproductive life history traits. Our predictions for this secondary analysis were that ecological constraints on fecundity should result in decreased fecundity under severe environmental conditions or when annual breeding occurs, while social constraints should manifest as a negative correlation between the number of mature females and average fecundity.

## Methods

### Study species

Hoary marmots are highly social arctic and alpine herbivores that live near patches of exposed talus scattered throughout the mountains of western Canada [13]. Unlike other alpine marmot species, hoary marmot family groups may exhibit both monogamous and polygynous mating strategies [16]. Although dominant females may suppress reproduction by subordinates or practice infanticide, multiple litters per family group, from multiple females, are common. Typically, family groups contain only one dominant male, who fathers all offspring. In our study site, extra-pair paternity was infrequent, and the timing of juvenile emergence suggests that most reproduction occurs prior to emergence from hibernacula in the spring [16].

### Study Site

This study was conducted on hoary marmots inhabiting a single 4 km^2^ valley in the southwest Yukon (61°12’N, 138°16’W; 1700–2100m). The valley is entirely above tree-line, and is characterized by a mix of wet and dry tundra interspersed with talus. Dominant plant species include *Dryas octopetala*, *Cassiope tetragona*, *Carex spp*., and a variety of dwarf willow species (*Salix spp*.) Hoary marmots, collared pikas (*Ochotona collaris*), and arctic ground squirrels (*Urocitellus parryii*) are the dominant herbivores, although caribou and Dall sheep are also present.

### Capture methods

From May to August, 1999 to 2004, we live-captured, marked and released most marmots in the population, Live-traps of various sizes (Tomahawk Live Trap Company, Tomahawk, WI) were baited with human urine [25] and live vegetation from the adjacent alpine meadows [26,27]. Juveniles were captured upon emergence from the natal burrow in early- to mid-July. At first capture, all marmots were marked in each ear using No. 3 monel tags (National Band and Tag, Newport, Kentucky) and a small piece of colored wire. A unique alphanumeric combination was dyed (Clairol Hydrience, #52 Black Pearl, Clairol Canada, Montreal, Quebec or Nyanzol-D American Colour and Chemical Corp., Charlotte, NC) into the fur above the tail to allow for individual identification at up to 200 m with binoculars. Colored wires were replaced annually, and faded dyed markings were reapplied as necessary at subsequent recaptures. Marmots were also re-sighted opportunistically by all field personnel throughout the summer, and their locations recorded according to a north-oriented grid marked at 50m intervals with wooden stakes.

### Ethics Statement

Kluane First Nations provided permission to conduct fieldwork on their land. All trapping procedures were approved by the University of Alberta Biosciences Animal Policy and Welfare Committee.

### Social group assignment

Most individuals were assigned to a social group based on observations of spring emergence from a common hibernaculum. If animals were not observed at emergence, we assigned social groups based on observation of social interactions and home-range overlap [16]. Individual home ranges were estimated using location data for resighted and radio-tagged marmots, which were analyzed in program Ranges V [28]. In a subset of marmots with known social group affiliations, group members had >75% overlap in the 95% kernel estimate of home range [29]. We therefore used 75% as a cutoff for statistical social group assignments. These assignments were then verified based on social interactions between individuals. Juveniles were assigned to the social group of their putative mother, which was subsequently verified using genetic markers [16]. Only marmots that could be confidently assigned to a social group in our study area (i.e. non-transients) were included in our analyses.

### Analyses

From an initial dataset of 217 marmots trapped between 1999 and 2004, we created a second, more limited dataset consisting only of female marmots two years old or older (76 individuals, 203 marmot-years). We excluded yearlings from this dataset because hoary marmots are not reproductively mature until age two, and generally do not reproduce until age three. In each year, we classified marmots as breeders or nonbreeders based on two criteria: genetic parentage assignment and evidence of lactation during capture. Finally, we generated an encounter history for each animal in which the individual was considered present if sighted or captured at least once that year.

In 2007–2009, a subset of four social groups was trapped, representing approximately half the population of the valley. All measurements and marking techniques were identical to those used in the earlier census. We could not definitively assign all litters to parents or determine the breeding status of all females in 2007–2009, so these data were excluded from our CMR analyses. However, the number of litters, and the number of juveniles per litter (within a week of emergence), were counted for all four social groups trapped in 2007–2009. Hoary marmot females produce one litter per year [16,30], so we used the number of litters as an estimate of the number of breeding females in those years. Data from 2007–2009 were used to qualitatively evaluate the ability of our CMR models to predict the number of breeding females in a given year, and were incorporated into models of fecundity and litter size.

Breeding histories from 1999–2004 were used to estimate breeding frequencies for each year and for the dataset as a whole. We used these data to compare breeding frequencies in the current year (2000–2004) between breeders and nonbreeders from the previous year. The null expectation, based on the hypothesis that biennial breeding does not occur, was that breeding frequencies in the current year should be independent of breeding state from the previous year.

### Multi-state CMR analysis framework

We analyzed the adult female mark-recapture dataset using multi-state models [22,23]. This involved constructing a set of candidate linear models to explain three parameters that jointly specified the probability of a female marmot being observed in either a breeding or non-breeding state. The three parameters were apparent survival (S), detection probability (p), and a ‘movement’ parameter describing the probability of moving between a ‘Nonbreeder’ state and a ‘Breeder’ state (ψ) [23]. Multi-state mark-recapture modeling allowed us to formally compare the likelihood of models representing our alternative hypotheses, including models where breeding state influenced future breeding state and survival simultaneously [23]. This analytical approach also allowed us to estimate the uncertainty associated with each parameter by accounting for variation between individual encounter histories. Because we were interested in breeding probability, our results only include ψ values reflecting the probability of transitions into the ‘Breeder’ state. Before analyzing the data, we conducted a goodness-of-fit (GOF) test using program U-CARE [31]. Because we did not detect significant lack of fit in a fully time- and group-dependent global model, we were able to use Akaike’s Information Criterion corrected for sample size (AICc) to compare models [32]. Based on previous analysis [24], p was modeled as a constant. The mean detection probability was 0.96 *±* 0.02 (SE).

### Candidate model set: Probability of breeding

We selected the models in our candidate model set to represent alternative hypotheses about the effects of age, social structure, climate, and previous breeding state on the probability of an individual breeding. Age effects were included because we expected that older, more experienced marmots would be more likely to reproduce than those which had recently matured. To account for the possibility that previous breeding state did not influence current breeding probability, we included some models where the probability of a mature female that bred last year also breeding this year (ψ _Breeder-Breeder_) and the probability of not breeding last year but breeding this year (ψ _Nonbreeder-Breeder_) were constrained to be the same. In models where previous breeding state was included, it was constrained to affect only older (> three years old) marmots, because two-year-olds generally do not breed. Only 2 two-year-olds reproduced during the course of the study, and these individuals were excluded to simplify analysis.

We used the mean value of the Pacific Decadal Oscillation index (PDO) from November and May to model winter climate (http://www.esrl.noaa.gov/psd/data/climateindices). PDO is a 20–30 year cyclic pattern of climate variation in the North Pacific Ocean that correlates well with temperature and precipitation throughout northwestern North America [33,34]. Within its multidecadal cycle, PDO also fluctuates annually. These annual fluctuations were negatively correlated with the date of spring snow-melt in an earlier study at our site [35], annual snow accumulation on nearby Mt. Logan (~100 km) [36], and mean winter snow depth at two weather stations (Burwash Landing and Aishihik Lake) each located ~30km away from our study site but 1400m lower in elevation (r = 0.61 and 0.6 respectively for 1967–2007; Environment Canada http://www.climate.weatheroffice.ec.gc.ca/). Positive winter PDO values are therefore associated with early snowmelt and shallow snowpack, while negative values are associated with the opposite patterns.

The social environment was measured as i) the total number of non-juvenile marmots within the social group that were resighted or captured after July 1 during the previous summer and ii) the number of reproductively mature females in the group. Both covariates should be negatively correlated with breeding probability if reproductive suppression is a common occurrence. A complete list of covariates used, as well as their abbreviations, is given in Table 1.

**Table 1:** Abbreviations and descriptions of covariates used in analyses of female hoary marmot reproductive parameters.

Abbreviation	Definition and Description
PDO	Mean Pacific Decadal Oscillation from November to May during the most recent winter
PDO_lag_	Mean Pacific Decadal Oscillation from November to May during the previous year
Age	Two age classes: Young (3 years old), and Old (>3 years)
Mother Age	Minimum age of mother in years, for litter size analysis only
Group	Total number of non-juvenile marmots within social group
Ad. fems	Number of reproductively mature adult females in a social group
Time	Random annual variation
Brd. State	Factor variable. 1 = females that bred during the previous year, 0 = nonbreeders in previous year
1	No time variation (constant)
Young	Denotes a linear covariate applied only to female marmots 3 years of age.
Old	Covariates applied only to female marmots >3 years old.

### Candidate model set: Apparent survival

Multi-state models included only three survival covariates: winter PDO, winter PDO lagged by one year, and current breeding state. Previous survival analyses suggested that winter climate was by far the dominant influence on survival; however, those analyses did not include breeding state. We therefore chose this simplified model set in order to test whether breeding significantly reduced the probability of surviving the following winter, while also accounting for the influence of winter climate and the timing of spring snowmelt. The two PDO indices were not correlated (r = 0.13, p = 0.85).

### CMR analyses / evaluation of fit

We constructed all mark-recapture analyses using program MARK and the RMark package in R [37,38]. We ranked models using AICc [39], and evaluated the relative importance of covariates by summing their AICc weights across the entire model set [32]. We also model-averaged Ψ and S across the entire model set [32,40]. Finally, we used the averaged Ψ values to predict the number of breeding females in 2007–2009. These predictions were compared with the estimated number of breeders from those years to test the generality of our results.

### Body condition index

We estimated body condition of captured adult female marmots using the residuals from a regression model of body mass at capture as a function of zygomatic arch width. The residuals from this model reflect body mass corrected for structural size, and are therefore expected to indicate animals with more than average energetic reserves relative to their skeletal size [41]. Body mass:skeletal size residuals are commonly used to indicate body condition in vertebrates, including alpine marmots [19,42,43] and are significantly associated with total fat content and lean mass in several rodent species [42]. We chose zygomatic arch width as a measure of structural size because it is a reliable indicator of age and sex in hoary marmots, outperforming other size measurements such as total body length and hindfoot length [44]. We tested whether breeding state had a significant effect on body condition over the course of the growing season by regressing body condition against breeding state, day of year, and an interaction term after log-transforming condition index values to meet the assumption of normality.

### Fecundity models

We used generalized linear mixed models (GLMM’s) to examine the effects of winter PDO, lagged winter PDO, group size, and group*climate interactions on three measures of hoary marmot fecundity. Models were fit using the lme4 package in R [45]. The three response variables were juveniles per social group (n = 78), average fecundity (juveniles per adult female w/in group; n = 66), and litter size using a dataset of all fully enumerated litters with known mothers identified by genetic analyses (n = 25) [16]. The litter size analysis also included models of litter size as a function of breeding history (annual vs. biennial breeding). We were able to fully enumerate 42 litters with genetically identified mothers, but could not determine the mother’s breeding history for 17 litters because the mother had not been detected or had been too young to breed in previous years. Error distributions were chosen after testing for conformity to a Poisson distribution [46]. Random effects were included based on likelihood ratio tests using the most parameterized fixed-effects model in each model set [47]. In all cases, we ranked models and calculated the relative support for individual variables using AICc. Beta-coefficients were model-averaged, and unconditional standard errors for those coefficients were estimated by bootstrapping with 10,000 replications. Only models that were within 7 AICc of the top model, and with smaller AICc values than an intercept-only (null) model were used for model averaging and for calculating AICc weights [32].

## Results

### Breeding frequency

54% of adult females who bred in the previous year also bred in the current year, while 47% of reproductively mature nonbreeders from the previous year bred during the current year (Table 2). The current-year breeding frequencies of breeders and nonbreeders from the previous year were not significantly different (Fisher’s exact test, p = 0.55). Current-year differences between previous-year breeders and nonbreeders were non-significant when each year was examined separately as well.

**Table 2:** Current versus previous-year breeding states.

		Breeding state in current year	
		N Number (%)	B Number (%)
Breeding state last year	N	27 (53)	24 (47)
	B	22 (46)	26 (54)

Contingency table comparing current and previous-year breeding states for adult female hoary marmots in the Ruby Range, Yukon Territory. For both the current and previous year, N = nonbreeder and B = breeder. Each cell shows the total number of females with a specific two-year breeding history. Percentages by row are shown in parentheses (e.g. Top row: % of previous-year nonbreeders who were nonbreeders or breeders in the current year). Breeders and nonbreeders from the previous year were equally likely to breed in the current year (Fisher’s exact test, p = 0.55). Test results were also non-significant when calculated separately for each year 2000–2004.

### Breeding probability

**Fig 1:**
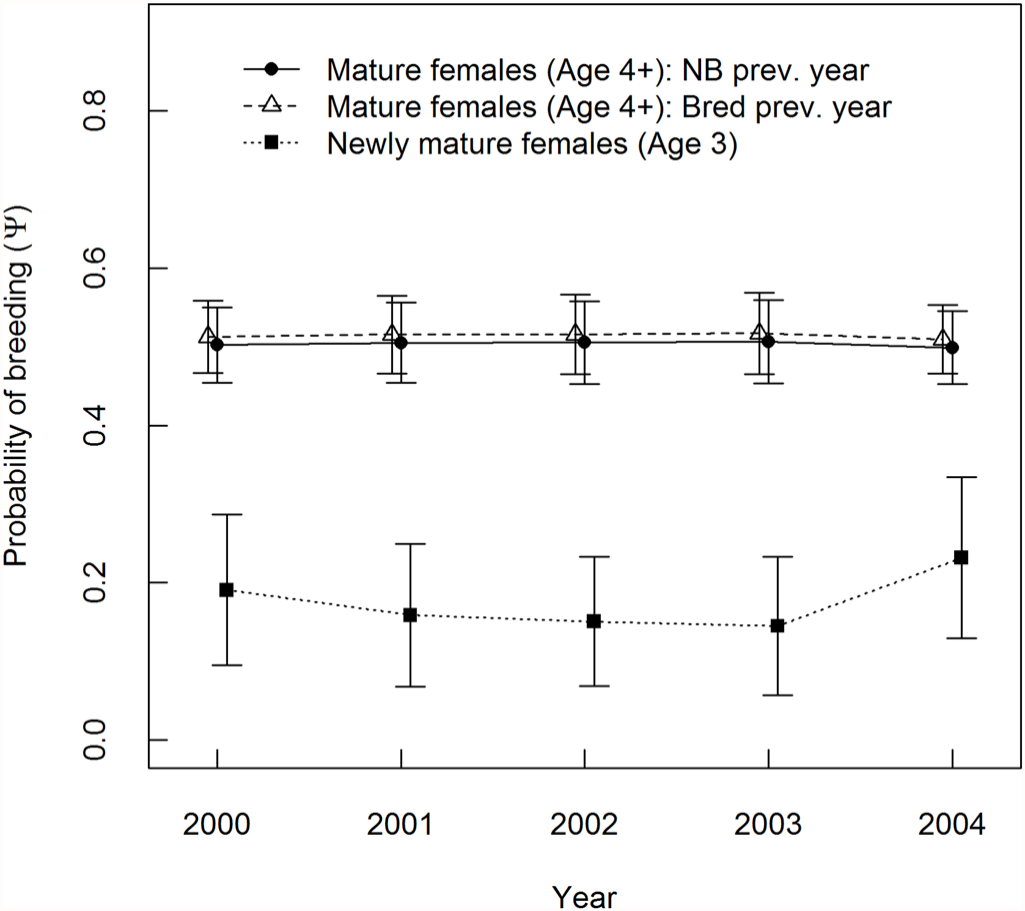
Model-averaged breeding probability. Probability of breeding (Ψ) for adult female hoary marmots was modeled as a function of age, previous breeding state, and time. Results are based on 6 years of trapping data (1999–2004) for marmots from 10 social groups in a single valley in the Ruby Range, Yukon. Values are model-averaged annual parameter estimates ± 1 SE.

For females who were reproductively mature during the previous year, the mean probability of breeding in the current year was 0.51 (SE = 0.05) for previous-year breeders and 0.50 (SE = 0.05) for previous nonbreeders (Fig. 1). The top model did not include previous breeding state as a covariate, but models with previous breeding state as a predictor of Ψ (the probability of moving into the ‘Breeder’ state) had a combined AIC weight of 0.48, and 3 models containing this covariate were within 2 AICc of the top model. Model selection therefore provided a moderate degree of support for effects of previous breeding state on current breeding effort (Table 3). However, the model-averaged effect size (difference in Ψ probability between breeders and nonbreeders from the previous year) was < 0.01 (Fig. 1). Model selection did not support PDO as a predictor of Ψ (Table 4). Social group size had a combined AIC weight of 0.28 (Table 4), but the Group covariate was only present in one model within 2 AICc of the top model. The best model did not include annual variation in breeding probability (Table 3), and model-averaged Ψ estimates varied by less than 1 SE from year to year in both age-classes (Fig. 1). Age-class had the strongest support of any model covariates, and had the largest effect size (Table 4; Fig. 1). Annual variation in Ψ was more pronounced for females in their first year of reproductive maturity, but the uncertainty in parameter estimates was also greater for this age-class (Fig. 1). On average, the breeding probability of females four years old or older was 0.33 greater than that of three-year-old individuals (Fig. 1).

**Table 3:** Model-selection results for linear models of hoary marmot reproductive parameters.

Model	K	AICc	Δ AICc	ω
S PDO + PDOlag	6	384.19	0	0.29
Ψ Age				
S PDO + PDOlag	8	385.28	1.09	0.17
Ψ Old: (Brd. State + Group)				
Ψ Young: Group				
S PDO + PDOlag	7	385.73	1.55	0.14
Ψ Old: Brd. State				
S PDO + PDOlag + Brd. State	7	385.93	1.75	0.12
Ψ Age				
S PDO + PDOlag + Brd. State	9	387.09	2.9	0.07
Ψ Old: Brd. State + Group				
Ψ Young: Group				
S PDO + PDOlag + Brd. State	8	387.51	3.33	0.06
Ψ Old: Brd. State				
Ψ Young: 1				
S PDO + PDOlag	6	387.91	3.73	0.05
Ψ Old: Ad. Fems				
Ψ Young: 1				
S (PDO + PDOlag) * Brd. State	9	388.71	4.53	0.03
Ψ Age				
S PDO + PDOlag + Brd. State	7	389.66	5.47	0.02
Ψ Old: Ad. Fems				
Ψ Young: 1				
S PDO + PDOlag	6	389.85	5.66	0.02
Ψ Old: Group				
Ψ Young: 1				
S (PDO + PDOlag) * Brd. State	11	389.97	5.79	0.02
Ψ Old: (Brd. State + Group)				
Ψ Young: Group				
S (PDO + PDOlag) * Brd. State	10	390.36	6.17	0.01
Ψ Old: Brd. State				
Ψ Young: 1				
S PDO + PDOlag	12	390.75	6.57	0.01
Ψ Old: Brd. State + PDO + PDOlag + Group				
Ψ Young: PDO + PDOlag + Group				

Model-selection results for multistate CMR analyses of adult female hoary marmot survival and breeding probability in the Ruby Range, Yukon Territory from 1999–2004. Mark-recapture data were used to model the joint probability of three parameters: Apparent survival probability (S), the probability of breeding in a given year (Ψ), and detection probability (p). p was always modeled as a constant, and was estimated at 0.96 ± 02 (SE). Models are described in terms of the covariates used to constrain S and Ψ. In some cases, Ψ was modeled differently for young (2 year old) and old (3+ years) marmots. Descriptions and abbreviations for all covariates are in Table 1. K is the number of estimated model parameters, AICc is the Akaike Information Criterion corrected for sample size, Δ AICc reflects the difference in AICc between each model and the top model (smallest AICc),and ω is the model’s AIC weight.

**Table 4:** Summed AIC weights for hoary marmot multi-state CMR analysis.

Covariate	ω+
**Ψ**	
Age	1
Brd. State	0.47
Age*Brd. State	0.47
Group	0.28
Age*Group	0.28
Ad. Fems	0.06
PDO	0.01
PDO_lag_	0.01
Age*PDO	0.01
Age*PDO_lag_	0.01
Age*Ad. Fems	0
**S**	
PDO	1
PDO_lag_	1
Brd. State	0.33
Brd. State*PDO,PDO_lag_	0.06

AIC weights (ω+) are shown for all covariates summed across all candidate models in a multi-state CMR analysis of adult female hoary marmots in the Ruby Range, Yukon, 1999–2004. Covariates of both survival (S) and breeding probability (ψ) are shown. Covariate descriptions are in Table 1.

‘*’ indicates an interaction term between two main effects.

When model-averaged parameters were used to predict the number of breeding females in a subset of the population during 2007–2009, the results were within 1–2 litters of the observed values in each year (Table 5). Year-to-year variation in the observed number of breeding females was also qualitatively similar to the predicted pattern (Table 5).

**Table 5:** Observed versus predicted number of breeding females.

Year	Predicted	Observed
2007	6	7
2008	8	10
2009	3	4

Observed and predicted number of breeding females summed across four hoary marmot social groups from the Ruby Range, Yukon, 2007–2009. Predictions were based on model-averaged breeding probability estimates derived from the same study site in 1999–2004, assuming one litter per breeding female per season. Predictions were rounded to the nearest whole number.

### Survival / body condition

**Fig 2:**
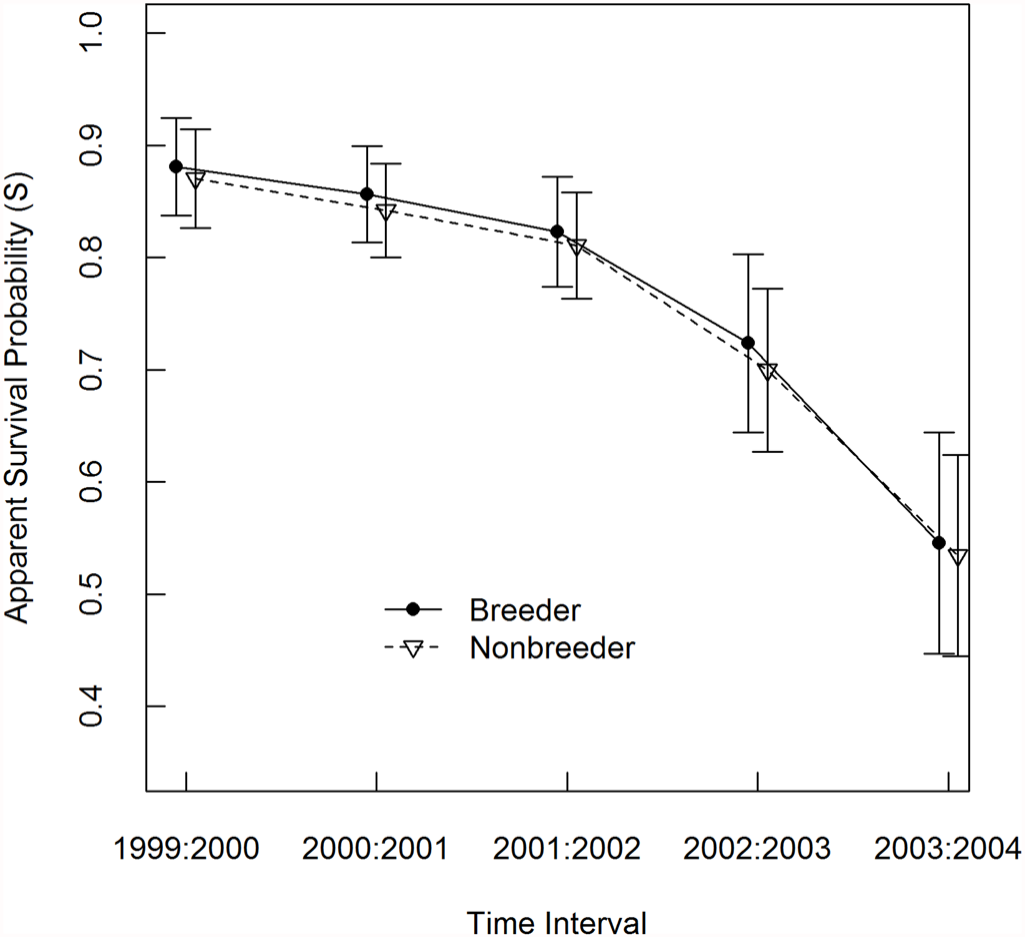
Model-averaged apparent survival probability. Apparent survival probability (S) was modeled for adult female hoary marmots in the Ruby Range, Yukon, between 1999 and 2004. Probabilities for breeding and non-breeding individuals are shown. Values are model-averaged parameter estimates ± 1 SE.

Breeding state had a summed AIC weight of 0.33 as a predictor of survival, but its effect size (difference in apparent survival probability) was less than 0.01 (Table 4; Fig. 2). In contrast, PDO and PDO lagged by one year were predictors of survival in all supported models (Table 3). PDO and PDO_lag_ were negatively correlated with survival probability, which declined over the course of the study by ~35% (Fig. 2).

**Fig 3:**
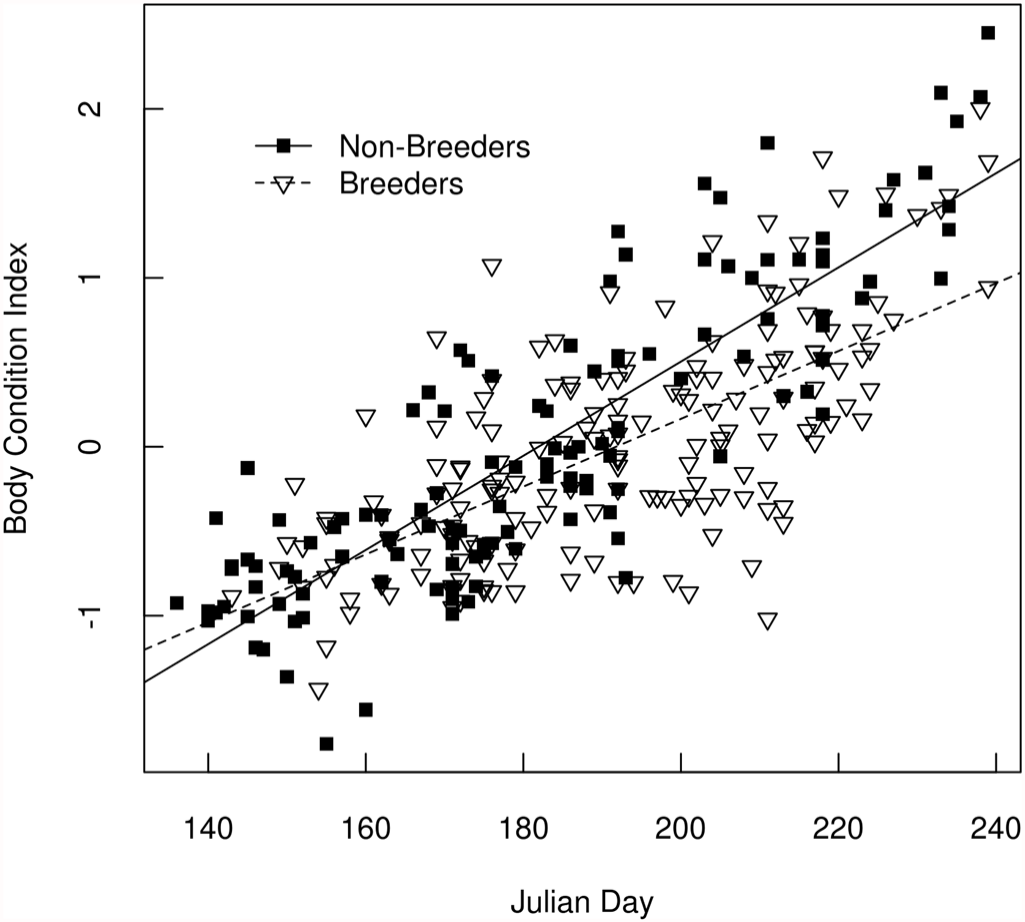
The Relationship between body condition and day of year for breeding and non-breeding female hoary marmots. We estimated the change over time in the body condition index (residuals from a linear regression of body mass as a function of zygomatic arch width) of non-breeding and breeding female hoary marmots in the Ruby Range, Yukon. Data from 1999–2004 are shown pooled across years. The best fit lines for linear regressions are shown. The slope of the relationship between body condition and Julian day was 0.02 (SE = 0.0016) for breeders and 0.028 (SE = 0.0015) for nonbreeders. The effects of day, breeding state, and day * breeding state interaction were all significant (p < 0.005) in a general linear model. Overall the model had an adjusted R^2^ of 0.62.

The body condition index of non-breeding females increased faster over the course of the summer than that of breeders (Fig. 3). The slope of the relationship between body condition and Julian day was 0.02 (SE = 0.0016) for breeders and 0.028 (SE = 0.0015) for nonbreeders. In a general linear model of body condition index as a function of Julian day, breeding state, and an interaction term, all three terms were highly significant (p<0.005). The overall model had an adjusted R^2^ of 0.62. On average, nonbreeders had higher body condition scores (greater body mass relative to skeletal size) at the end of August than breeders (Fig. 3).

### Group fecundity

The juveniles per group data set contained significant Poisson overdispersion, so negative binomial models were used (Table 6). Likelihood-ratio tests did not support either slope or intercept random effects (Table 6). Social group size was a predictor in the top model and in seven of the 10 other top-ranked candidate models, and had a summed weight of 0.79 (Table 7, Table 8). Each additional group member corresponded to ~1 additional juvenile (e^0.06^ = 1.06; Table 8). There was weaker support for a relationship between juveniles per group and winter PDO. PDO had a summed AIC weight of 0.4, and PDO_lag_ had a summed weight of 0.31 (Table 8). PDO was negatively correlated with the number of juveniles produced per group (Table 8). The bootstrap 95% confidence interval for the PDO beta coefficient was large and overlapping with 0 (Table 8). The model-averaged effect of PDO_lag_ on juveniles per group was positive, but the 95% confidence interval for that coefficient overlapped with 0.

**Table 6:** Fecundity data Poisson overdispersion tests.

Response Variable	Obs./Theor. Var	Statistic	p	Error Distribution	Random Effects
Juveniles/Group	3.83	248.64	0	Neg. Binomial	None
Juveniles/Female	1.29	84.14	0.06	Poisson	Social Group, Social Group*PDO
Known Litter Size	0.57	23.33	0.99	Gaussian	None

Results of Poisson overdispersion tests [44] for three measures of hoary marmot fecundity, based on data from 10 hoary marmot social groups in the Ruby Range, Yukon Territory, 1999–2004 and 2007–2009. The corresponding linear model types used to model those variables are also shown. Error distributions were chosen based on test results. If significant overdispersion was present, negative binomial generalized linear models were used. If Poisson underdispersion was detected, Gaussian errors were used. Random effects (Social Group affiliation, and interaction between Social Group interaction and fixed effect slopes) were included based on likelihood-ratio tests using the most parameterized fixed-effect model in each model set [45].

**Table 7:** Model-selection results for linear models of hoary marmot reproductive parameters.

Model	K	AICc	Δ AICc	ω
Juveniles Per Group				
(*Negative Binomial GLM’s*)				
Group	3	315.63	0	0.22
Group+PDO	4	315.88	0.25	0.19
Group+PDO_lag_	4	316.12	0.48	0.17
Group+PDO+PDO_lag_	5	316.55	0.92	0.14
Group*PDO	5	317.91	2.28	0.07
Null	2	318.33	2.69	0.06
PDO	3	318.35	2.71	0.06
Group*PDO_lag_	5	318.46	2.82	0.05
PDO_lag_	3	320.45	4.82	0.02
PDO+PDO_lag_	4	320.56	4.93	0.02
Group*(PDO+PDO_lag_)	7	320.87	5.24	0.02
Juveniles Per Female				
(*Poisson GLMM’s*)				
PDO+PDO_lag_	4	185.04	0	0.38
Group*(PDO+PDO_lag_)	7	186.98	1.94	0.14
Group+PDO+PDO_lag_	5	187.23	2.19	0.13
Group*PDO	5	187.32	2.28	0.12
PDO	3	187.66	2.62	0.1
Group+PDO	4	188.34	3.3	0.07
PDO_lag_	3	189.87	4.83	0.03

Data were collected 1999–2004 and 2007–2009 from 10 social groups in the Ruby Range, Yukon Territory. K is the number of estimated model parameters and ω is the AIC weight. Response variables were the number of juvenile produce per social group and average fecundity (juveniles per female within social group). The error distribution used and the type of model are shown in italics below the name of each response variable. GLMM’s are Generalized Linear Mixed Models, and GLM’s are Generalized Linear Models. Only models with Δ AICc < 7 are shown.

**Table 8:** Model-averaging results for fecundity linear models.

Model	Model-averaged β	SE	LCL	UCL	ω+
Juveniles Per Group					
(*Negative Binomial GLM’s*)					
PDO	-0.13	0.36	-1.24	0.03	0.4
PDO_lag_	0.09	0.19	-0.17	0.58	0.31
Group	0.06	0.04	0	0.14	0.79
Group*PDO	0	0.03	-0.02	0.12	0.07
Group*PDO_lag_	0	0.02	-0.02	0.05	0
Juveniles per female					
(*Poisson GLM’s*)					
PDO	-0.26	0.49	-1.69	0.17	0.94
PDO_lag_	0.2	0.37	-0.5	1.03	0.68
Group	0	0.04	-0.08	0.09	0.46
Group*PDO	0.01	0.05	-0.05	0.16	0.26
Group*PDO_lag_	0	0.05	-0.11	0.1	0.14
Litter Size					
(*Gaussian GLM’s*)					
Intercept	3	0.19	2.64	3.38	1

Model-averaged beta coefficients (β), bootstrapped unconditional standard errors (SE), 95% confidence interval lower and upper limits (LCL and UCL), and summed AIC weights (ω+) of parameters for models of group fecundity, individual fecundity (juveniles/adult female), and litter size of hoary marmots in the Ruby Range, Yukon Territory. SEs and 95% confidence limits were bootstrapped with 1000 replications. Except for litter size, analyses were based on data from 1999–2004 and 2007–2009. Due to highly significant poisson overdispersion, juveniles per group were modeled using negative binomial linear models. Juveniles per female were modeled using Poisson mixed models with social group random effects. Litter size data were approximately normally distributed, and were modeled as such. Only models that were within 7 AICc of the top model, and with smaller AICc values than an intercept-only (null) model were used for model averaging and for calculating AICc weights [32].

### Juveniles per female (average fecundity)

Average fecundity data conformed to a Poisson distribution, but likelihood-ratio tests supported social group as a random effect on the model intercept and the slope of the response to winter PDO (Table 6). Model selection strongly supported a negative relationship between average fecundity and winter PDO and a positive relationship with PDO_lag_ (Table 7, Table 8). However, the 95% confidence interval for the model-averaged PDO and PDO_lag_ beta coefficients overlapped with zero (Table 8). Social Group effects had a summed AIC weight = 0.46, but did not show a consistent negative or positive relationship with fecundity (Table 8). Interactions between climate and social effects were not as well supported as either main effect, and the confidence intervals for their beta coefficients all overlapped with zero.

### Litter size

No litter size model with breeding history as a predictor outperformed the null (intercept-only) model. We therefore re-ran the litter size analysis using all 42 fully enumerated litters, but excluding models that included breeding history. When we did so, four models had lower AICc values than the null model, but all were within 2 AICc, indicating that the null model provided comparable predictive power. The mean litter size was. Using the larger dataset, the probability of a type II error was low. For example, for a model with litter size as a function of PDO alone, power associated with an effect size (Cohen’s f^2^) of 0.33, which corresponds to an R^2^ of 0.25 [42], was 0.95. Litter size ranged from one to six individuals, although these extreme values were rare. The mean litter size was 3.0 (SE = 0.19; Table 8). Average litter size did not vary significantly between social groups (F_9,41_ = 0.61, p = 0.78).

## Discussion

Hoary marmots are not obligate biennial breeders. Although a mature female marmot’s probability of breeding was close to 50% on average, none of the three hypotheses we examined (Evolved Constraints, Ecological Constraints, and Social Constraints) could adequately explain variation in breeding probability.

### Evolved Constraints

Contrary to the predictions of the Evolved Constraint hypothesis, breeding in the previous year had almost no effect on the probability that an individual would breed in the current year. Although hoary marmots do not appear to have evolved to be obligate biennial breeders, the fact that average breeding probability was well below 100%, without correlating to environmental or social variables, leads us to hypothesize that their reproductive success may still be influenced by evolved physiological constraints. Life history theory predicts that life history parameters with the greatest potential influence on population growth should be the least variable [48]. Hoary marmots may therefore have evolved a consistent probability of breeding because breeding probability is more important, demographically, than other aspects of their life history. This hypothesis could be tested by estimating the sensitivity of population growth rates to breeding probability compared to other demographic parameters.

### Ecological Constraints

Breeding probability was not sensitive to the depth and duration of snowpack the previous winter or to the length of the previous growing season as indexed by the winter PDO. During the study period, PDO had a strong effect on survival, so the lack of evidence for ecological constraints on breeding probability (Fig. 2) is probably not attributable to mild winter conditions [24]. However, our results may reflect a life-history trade-off in which constant breeding effort is necessary to balance the mortality risk associated with a harsh and variable environment, since winter conditions did have a strong influence on survival. A stable probability of breeding may also have come at the expense of fecundity. This last hypothesis is consistent with our fecundity model selection results, which showed a weak trend for average fecundity to decline during winters with thin snowpack (high PDO values) while breeding effort did not. Finally, breeding after particularly harsh winters may result in maternal stress effects being passed on to juveniles [49]. We cannot test this hypothesis directly, but juvenile hoary marmot survival is correlated with winter climate lagged by one year, which implies the operation of maternal effects [24].

### Income or Capital breeding?

The seasonal activity patterns of breeding adult females in our study were consistent with previous research [13], and breeders were at an energetic disadvantage by the end of the season (Fig. 3). However, this difference had no measurable impact on either apparent survival probabilities or on the probability of breeding again the following year. These results suggest that hoary marmots are income breeders, and are able to adequately recover the physiological costs of reproduction (i.e. acquire sufficient income) during the current growing season [17]. Our study population appeared not to be food limited, since breeding probability was not related to growing season length even though the growing season varied by almost four weeks from year to year [35]. This conclusion is bolstered by the results of a food addition experiment at our study site, in which marmots fed rabbit chow *ad libidum* did not show significant differences in mass accumulation rates, fall body mass, or overwinter survival compared to control animals (T.J. Karels et al. unpublished data).

### Social Constraints

Our results did not support the Social Constraint hypothesis. Had partial reproductive suppression occurred, breeding probability or the average number of juveniles per female should have been negatively correlated with group size, which we did not observe [4]. Instead, social group size had little influence on average fecundity, and was positively related to the number of juveniles born per group in a given year. When social structure was modeled using the number of adult females as a covariate instead of total group size, the results were essentially the same. Finally, although the observed effect of age on breeding probability could reflect partial reproductive suppression if older females were more likely to be dominant, social mechanisms are not required to explain a link between age and reproduction [19,21,50]. Hoary marmot breeding probability may be poorly constrained by social factors because females within a social group are closely related. In alpine marmot, the number of dominant females who breed is negatively related to the number of subordinate females present, but this effect is weakest when subordinates are offspring of the dominant female [19]. Although genetic relatedness has not been comprehensively determined in our population, hoary marmot females rarely disperse from their natal group, and extra-pair copulation is almost non-existent [16], so females within a social group are mostly offspring, grand-offspring, or siblings of each other. In addition, the number of adult females per group is highly variable [16], so the social dominance structure must also be unstable. Hoary marmot females may receive enough direct and indirect fitness benefits that reproductive competition is unnecessary.

### Biennial breeding revisited

There are several potential explanations for the contrast between our findings and previous literature. It is possible that our study population was not subject to environmental or social constraints severe enough to impose biennial breeding. However, in our study the date of spring snowmelt varied by several weeks, and social groups ranged in size from 2 to ~30 individuals [16,24]. In addition, our study site was similar in latitude and habitat to the area where Holmes [51] reported obligate biennial breeding in hoary marmots. We believe it is more likely that earlier studies of hoary marmot breeding by Barash [14] and Holmes [51] relied on inadequate sample sizes (n = 9 in both cases) and study durations (one year and three years, respectively). In such circumstances, an average breeding probability close to 50% could easily be mistaken for a biennial pattern. Vancouver Island marmots and Olympic marmots were also initially described as biennial breeders based on limited sample sizes (n = 6 and 9 females, respectively) [52,53]. In both cases, later studies suggested that apparently obligate biennial breeding may actually be a facultative response to environmental constraints [15,54]. Similarly, King Penguins (*Aptenodytes patagonicus*) were described as biennial breeders based on a 14 month study [55] but their breeding patterns are now understood to be variable and constrained by winter food availability [1]. Even long-term studies can yield misleading conclusions about biennial breeding patterns. For example, a population of mountain goats studied for two decades [3] exhibited near-universal biennial breeding for 16 years before switching to annual breeding as a result of declines in population size (and presumably reduced intraspecific competition for food). Distinguishing obligate from facultative biennial breeding is difficult, and requires careful analysis of breeding probabilities across a broad range of environmental and/or social conditions.

As the preceding examples suggest, ecological constraints are the most common drivers of facultative biennial breeding, particularly in mammals. When ecological constraints are strong and affect entire populations simultaneously, they can easily be mistaken for obligate patterns [56]. In contrast, social constraints have only been linked to biennial breeding *per se* in the bigamous hoary marmot populations studied by Holmes [51]. Even this instance may essentially reflect ecological constraints, since the dominant female was reported to skip additional years when snowmelt was exceptionally late [57].

Although many species may skip breeding opportunities due to ecological constraints, biennial breeding as an evolved, obligate trait appears to be extremely rare even in arctic/antarctic/alpine animals. In mammals, the only compelling case of obligate biennial breeding that we are aware of is the walrus (*Odobenus rosmarus*), an arctic marine mammal with a 15-month gestation period [58]. Obligate biennial breeding in birds appears similarly confined to relatively high-latitude marine species like albatrosses and petrels, particularly in species with relatively large body sizes and long chick-rearing periods [2,5]. In both birds and mammals, obligate biennial breeders also have fixed, small litter or clutch sizes, which means they cannot accommodate the energetic costs of breeding by reducing reproductive output in bad years [5,58]. In contrast, alpine-dwelling marmots have variable litter sizes, and have average gestation and weaning times of 32 and 36 days, respectively, which means that females are typically free to spend half the growing season feeding themselves rather than their offspring [21]. These life history traits may explain why biennial breeding is not an obligate trait in marmots. Even albatross species which almost never breed annually can do so if their first chick dies early in the season, which further illustrates the rarity of obligate biennial breeding as an intrinsic, evolved trait [59].

## Conclusions

Hoary marmots, which have been described as obligate biennial breeders for four decades, are capable of annual breeding under variable environmental and social conditions. The flexibility of hoary marmot breeding patterns may be explained by an income-oriented breeding strategy that allows them to recover the energetic costs of breeding during the subsequent growing season. Our study demonstrates the inadequacy of average breeding frequency as an indicator of biennial breeding, and highlights the need for long-term datasets and robust analytical methods to evaluate hypothesized drivers of breeding probability. Of the three hypotheses we evaluated, ecological constraints are probably the most common drivers of biennial breeding patterns in birds and mammals. The effects of ecological constraints have frequently been mistaken for obligate biennial breeding driven by evolved constraints, which is a rare phenomenon. Though commonly ignored in demographic studies, breeding probability can have a strong influence on population growth rates [48]. Accurate characterization of breeding probability and its drivers is therefore essential for predicting population trajectories and assessing the vulnerability of species to climate change and other environmental stressors [60,61]. The evidence for biennial breeding and other unusual life-histories in arctic/alpine/antarctic species, particularly mammals, should therefore be re-examined.
